# Hermetic Bags Help Afghan Rural Women Preserve Wheat Flour during Winter

**DOI:** 10.3390/insects13030237

**Published:** 2022-02-27

**Authors:** Dieudonne Baributsa, Ibrahim B. Baoua

**Affiliations:** 1 Department of Entomology, Purdue University, West Lafayette, IN 47907, USA; 2 Département des Sciences et Techniques de Productions Végétales, Université Dan Dicko Dankoulodo de Maradi, Maradi BP 465, Niger; baoua.ibrahim@gmail.com

**Keywords:** wheat flour storage, insect infestation, quality loss, hermetic bags, Afghanistan

## Abstract

**Simple Summary:**

Afghan women face enormous challenges storing wheat flour during the winter due to high relative humidity and insect pests. These challenges result in poor-quality baking wheat flour. We conducted an experiment in Herat province, Afghanistan, to assess whether hermetic bags would preserve wheat flour during the wintertime when relative humidity is high. Forty women from two districts participated in this experiment. Each woman stored 25 kg of wheat flour in a Purdue Improved Crop Storage (PICS) bag and a polypropylene (PP) woven bag. The results showed that the moisture content, color, and baking properties of wheat flour stored in PICS bags for six months did not change. No insects were observed in wheat flour stored in PICS bags. However, wheat flour stored in PP bags had high moisture content and insect pests. The price of wheat flour stored in PICS bags increased over time, and that stored in PP bags decreased. Hermetic bags can be used to safely store wheat flour for six months in areas with high relative humidity conditions. Storing wheat flour in hermetic bags would help improve food security for millions of wheat consumers in developing countries.

**Abstract:**

On-farm preservation of wheat flour is a challenge due to insect pests and high relative humidity. This experiment was conducted to assess the effectiveness of hermetic bags in preserving wheat flour stored by women during the wintertime when relative humidity is high. Forty women (households) from two districts in Herat province, Afghanistan, stored their wheat flour for 6 months. Each woman stored 25 kg of wheat flour in a Purdue Improved Crop Storage (PICS) bag and a polypropylene (PP) woven bag. Moisture content, insect population, flour color, bread taste, and profitability of storage were assessed. Moisture content and insect population significantly increased in PP bags after six months of storage, while no changes were observed in PICS bags. There was a significant negative correlation between wheat flour color and moisture content (r = −70.7, *p* < 0.001) and insect population (r = −79.9, *p* < 0.001). Wheat flour stored in PICS bags for 6 months retained its color and produced better bread than that stored in PP bags. Storing wheat flour in PICS bags for six months showed a return on investments (ROI) of +16.9% against −33% for the PP bag. Farm households and other wheat value chain actors can safely store wheat flour in hermetic bags for up to six months under high relative humidity conditions. This would help improve food security for millions of wheat consumers in developing countries.

## 1. Introduction

Wheat is an important staple crop that occupies 70% of the cultivated land area and is the primary source of nutrition for millions of Afghans [1,2]. Wheat production is variable and mostly below its potential due to changing weather patterns and insecurity [3]. In 2013, total wheat production amounted to about 4 million tons, which fell short of the estimated 6 million tons needed to sustain the population [4]. Low wheat production combined with a growing population forces the formerly self-sufficient Afghanistan to rely on imports for its wheat supply [2]. Additionally, the shortage of wheat is linked to poor postharvest management. As a result, an estimated 44% of households perceived themselves to have some degree of food insecurity [5]. 

The postharvest management of stored products is a challenge among smallholder farmers in developing countries including Afghanistan [6]. Farmers lose substantial quantities of wheat grains and flour to insects, rodents, and mold due to poor postharvest handling and storage. Wheat flour losses can reach up to 30% during storage [7]. Studies have assessed the use of different storage methods in preserving wheat flour quality, including airtight and non-airtight containers, cold plasma, and traditional storage [8,9,10]. However, most of these methods are not effective, unaffordable, not available, or not applicable to the conditions of smallholder farmers. Assessing the use of commercially available hermetic bags for wheat flour storage is critical. 

In Afghanistan, women play an important role in wheat postharvest activities, including harvesting, threshing, storage, and processing [11]. Securing wheat flour during the wintertime (lean season) is critical to ensure households are food secure. Hence, wheat flour must be properly stored to preserve nutritional and baking properties. However, storing wheat flour during the winter in Afghanistan poses several challenges due to the increase in relative humidity. High humidity affects the quality of wheat flour, including moisture content, nutrient content, and baking properties [8,9,12]. In addition, insects pose major challenges during wheat flour storage and lead to significant losses [10,13]. 

Commercially available hermetic bags (e.g., Purdue Improved Crop Storage—PICS) are viable and cost-effective approaches that could help households preserve the quality of wheat flour. Hermetic storage works by depriving insects of the oxygen needed for metabolic activities [14]. PICS bags, disseminated in Afghanistan, have proven effective in maintaining the quality of stored legumes and cereal crops [15]. Several laboratory experiments have shown that hermetic containers such as plastic bottles and polyethylene bags can preserve wheat flour for several months [16,17]. No studies have looked at on-farm storage of wheat flour using commercially available hermetic bags. This study aimed to assess the effectiveness of PICS bags for on-farm storage of wheat flour. The results would be useful to households and other value chain actors who store wheat flour to improve food security.

## 2. Materials and Methods

### 2.1. Study Site and Treatments

This study was implemented in Herat province in Afghanistan from 29 November 2013, to 29 May 2014. The period corresponds to the wintertime, with an average rainfall of 241 mm, average relative humidity of about 40% to 80%, and temperatures varying from −1 to 12 °C in December and 13 to 29 °C in May [18]. 

Forty women (representing 40 households) from 10 villages in two districts (Zinde Jan and Injil) were involved in this on-farm experiment. The women were selected based on their willingness to participate and make 50 kg of wheat flour available for the duration of the experiment. Wheat flour was stored in two types of bags of 50 kg capacity—Purdue Improved Crop Storage (PICS) and polypropylene (PP) woven (control) bags. Each woman stored 50 kg of wheat flour—25 kg in a PICS bag and the other 25 kg in a PP bag. Only flour from wheat harvested during the 2013 cropping season was used in this experiment. Grain used to grind the flour was not treated with any insecticides. Each layer of the PICS bag was tied separately, following the standard procedure [19]. Polypropylene bags were tied using a rope as well. Each woman kept her PICS and PP bags in the house on an elevated platform (e.g., pallet or pieces of wood). A total of 80 bags were stored by these women, of which 40 were PICS bags and the other 40 were PP bags. The experiment was treated as a randomized complete block design with the blocking at the village level. 

### 2.2. Data Collection

Flour quality was assessed before the experiment and after three and six months of storage. Data were collected on several parameters, including insect infestation, flour color, moisture content, sensory evaluation of the baked bread, and price of flour. 

Insect infestation: Six samples of wheat flour, each weighing 250 g, were removed from each bag. Two samples were removed from the top layer, after which half of the flour was removed from the bag, and an additional 2 samples were collected. Finally, three-quarters of the flour was removed, and another 2 samples were collected. Each sample was sifted using a no. 25 sieve, and the number of live and dead insects were counted and recorded for each bag. All non-sifted flour was returned to the bag and sealed. 

Flour color: To assess the flour color, a score was used: a scale from 1(bad) to 5(excellent). A score of 5 represented flour in pristine, white condition, whereas a score of 1 represented a sample that had become thoroughly discolored and appeared dark or gray to the observer. The 250 g sample used for insect assessment was also used to assess flour color. The flour color was assessed by a panel of the four women from each village who participated in this experiment, but the samples were blinded after collection to avoid any biases. Each participant had the opportunity to assess flour stored in PICS and PP bags. 

Flour moisture content: Wheat flour moisture content was assessed via water loss by drying. Samples of 100 mL were removed from each bag and weighed on a scale. Then, all samples were placed in an oven to dry for 24 h. After the drying period, the samples were re-weighed, and percent moisture content was determined by the equation

$$\mathrm{MC}=\frac{M1-M2}{M1}$$

where M1 was the initial mass of the 100 mL sample of flour, and M2 was its final mass. Final moisture content values were recorded as percentages of the total mass.

Sensory evaluation of baked bread: A 0.5 kg sample of wheat flour was removed from each bag and taken to a baker. Baked bread was assessed by a panel of the four women from each village who participated in this experiment, but the samples were blinded to avoid any biases. Each participant had the opportunity to taste bread made with flour stored in PICS and PP bags. The bread taste was scored on a binary scale as either “Good” or “Not Good”. Bread designated as “Good” (scored as 1) indicated products that were deemed edible and could be sold at the local market. Bread designated as “Not Good” (scored as 0) indicated products that were determined to be inedible or otherwise could not be sold at the market. An average score of 1 indicated no change in quality from the initial product during storage. An average score of 0 represented the total rejection of all samples.

Price of flour: The price of wheat flour was collected from the nearest local market by blindly asking potential buyers how much they were willing to pay. These price data were collected at the beginning of the experiment, and then after three and six months of storage. Values were assigned in the local currency (Afghani) and listed as Afghanis/kg of wheat flour.

### 2.3. Data Analysis

ANOVA and LSD tests were used to compare moisture content and insect pest infestations between treatments at different observation periods. ANOVA was also used to assess the effect between the dependent and independent variables. Data were analyzed using SPSS version 26. Estimates of the return on investments (ROI) were computed using wheat prices at the beginning of the experiment, and then after three and six months.

## 3. Results

### 3.1. Significance of the Effect between Independent and Dependent Variables

Significant effects were observed between storage methods (PICS and PP bags) and the number of live and dead insects, and between storage methods and moisture content (Table 1). The price of wheat flour was significantly different between storage methods and among villages. However, no differences were observed among households, nor between the two districts on all variables. 

### 3.2. Moisture Content and Insect Infestation of Wheat Flour during Storage

The moisture content of wheat flour stored in PICS for 6 months did not increase (Table 2). However, the moisture content of wheat flour stored in PP bags increased by 1.00% and 3.00% after 3 and 6 months of storage, respectively (Table 2). *Tribolium castaneum* (Herbst), (Tenebrionidae, Coleoptera) was the only species recorded in the wheat flour. No live or dead insects were observed at the beginning of the experiment or in the PICS bags after 3 and 6 months of storage (Table 2). In PP bags, however, live and dead *T. castaneum* adults were observed in wheat flour after 3 and 6 months of storage. Moisture content had a significant positive effect on the development of the insect population in PP bags, with a Pearson correlation coefficient of 76.1 (*p* < 0.001).

### 3.3. Quality and Sensory Assessments 

The quality (i.e., color and taste) of wheat flour stored in PP bags for 6 months was significantly reduced, whereas it remained the same in PICS bags (Table 3). A little over half of the respondents noted that the color of wheat flour stored in PP bags for 6 months was not good, whereas 45% said that the color was fair. There were negative correlations between wheat flour color score and moisture content (r = −70.7, *p* < 0.001), live insects (r = −79.9, *p* < 0.0010), and dead insects (r = −65.7, *p* < 0.001). Unlike the PP bags, the color of wheat flour stored in PICS bags for six months received “good or excellent” scores by all the participants (Table 3). About three-fourths of the tasters indicated that the bread made with wheat flour stored in PICS bags for 6 months was excellent. However, the same number of participants noted that bread made with wheat flour stored in PP bags for six months was “not good”. 

### 3.4. Storage Profitability of Wheat Flour 

Wheat flour stored in PICS bags fetched a higher price—an increase of 16.40% after 3 months and 32.28% after 6 months (Table 2). The value of wheat flour stored in the PP bag increased by 2.91% after 3 months but dropped by 24.87% after 6 months (Table 2). There was a positive correlation between the price and the color quality of wheat flour (r = 72.0, *p* < 0.001). However, negative correlations were observed between the price of wheat flour and moisture content (r = −67.9, *p* < 0.001), number of live insects (r = –82.8, *p* < 0.001), and number of dead insects (r = −73.9, *p* < 0.001). The return on investments (ROI) varied between storage methods and was positive for PICS bags, whereas it was negative for the PP bags (Table 4). This net return for storing wheat flour in 100 kg PICS bags was about USD 2 in 3 months and USD 5.65 after 6 months. Storing wheat in PP bags would result in losses of USD 0.68 in 3 months and USD 10.5 in 6 months. 

## 4. Discussion

### 4.1. Effect of Moisture Content on the Quality of Wheat Flour during Storage

The moisture content of wheat flour stored in PP bags for six months increased significantly—by 36.2%—but no changes were observed in PICS bags. The storage conditions influenced the moisture content of wheat flour stored in PP bags, hence its quality. Relative humidity in Herat, Afghanistan is high during the winter months [18]. The hygroscopic properties of wheat flour are well known and change with variations in temperature and humidity of storage environments [16]. The high r.h. during the storage period may explain the increase in moisture content. Moisture content influenced the quality of the wheat flour stored in PP bags but not in PICS bags. The increase in moisture content of wheat flour causes biochemical and enzymatic reactions, leading to the deterioration of crude proteins and fats [8,12]. The high moisture content of wheat flour facilitated the proliferation of insects, as suggested by the strong positive correlation between both variables.

### 4.2. Impact of Pest Infestation on Wheat Flour Quality during Storage 

The wheat flour stored in PP bags was infested by *T. castaneum,* and the population increased with storage time. *Tribolium castaneum* is well known as a pest of wheat flour stocks [10,13,20]. The development of insects in PP bags was due to favorable environmental conditions such as high humidity [21,22]. Through its feeding activities, *T. castaneum* affects the quality of the wheat flour, including fatty acids, proteins, and gluten. The presence of *T. castaneum* can also result in the development of toxins such as benzoquinones and aflatoxins [13,20]. In addition, parts of dead insects can affect the quality of the wheat flour by increasing its water content, microbial fauna, and uric acid content, as well as changing the color of the flour [23]. These changes in physical and biochemical properties induced by the high level of humidity and the presence of insects explain the degradation of the organoleptic qualities of the wheat flour stored in PP bags for six months and its lower sensory scores among Afghan women. 

### 4.3. Efficacy of Hermetic Storage in Preserving Wheat Flour Quality

PICS bags maintained wheat flour quality during the six-month storage period. Hermetic bags including PICS are known to preserve the quality of grain and wheat flour during storage [17,21,24]. In a PICS bag, the presence of the two liners inside a PP bag prevents the exchange of humidity and air between the stored products inside the bag and the outside environment. Hermetic bags are known to maintain the initial relative humidity of stored products for several months and to prevent insect infestations and the accumulation of aflatoxins [16,17,24,25]. Similar results were observed during hermetic storage in Mozambique and Portugal, where insect infestation was reduced and the quality of paddy rice preserved [26,27]. The ability of PICS bags to maintain a constant relative humidity inside the bags and prevent the development of insect pests allowed the preservation of the initial organoleptic properties of the wheat flour stored for six months. This explains the high scores obtained from the panelists on the color and the taste of bread made using wheat flour stored in PICS bags for up to 6 months. 

### 4.4. Storage Profitability of Wheat Flour

After 6 months of storage, compared with the initial price, wheat flour price per kg increased by 16.4% when stored in PICS bags, whereas it decreased by 24.87% when stored in PP bags. Storing wheat flour in PICS bags resulted in a net return of AFA 339.3 or USD 5.66 per 100 kg, whereas in PP bags, farmers lost AFA 637.6 or USD 10.63. Using PICS bags to store wheat grain in Afghanistan is more profitable than using regular PP bags [24]. PICS bags are profitable in storing legume and cereal crops (e.g., cowpea, corn, and beans), with ROI varying between 13 and 80% [28]. The importance of wheat as a security crop in Afghanistan cannot be underestimated. Though the ROI is relatively low compared to some high-value crops, most wheat flour was stored for home consumption [29]. Securing good-quality wheat flour during winter times is critical for food security for millions of Afghan households.

## 5. Conclusions

This study shows that the PICS bag can preserve wheat flour during the wintertime when relative humidity is high. Wheat flour quality was minimally affected when stored in PICS bags. However, wheat flour stored in PP bags had high moisture content and insects, resulting in poor-quality bread. Using hermetic bags (e.g., PICS bags) would help millions of households become food secure by storing wheat flour for home consumption when humidity is high. Value chain actors involved in processing or storing wheat flour can use hermetic bags to preserve its quality.

## Figures and Tables

**Table 1:** The significance of the effect of storage methods, household, village, and district on the number of live and dead insects, moisture content, and price of wheat flour (Afghani, AFA) during wheat flour storage for six months in Herat Afghanistan.

	Insects		Moisture Content	Price of Wheat Flour
	Alive	Dead		
Storage methods	***	***	***	***
Household	NS	NS	NS	NS
Village	NS	NS	NS	*
District	NS	NS	NS	NS

*, *** Difference among regions significant at levels 0.05, and 0.001, respectively; NS: no significant difference.

**Table 2:** Moisture content, insect infestation, and price (Afghani, AFA) of wheat flour stored in Purdue Improved Crop Storage (PICS) and polypropylene (PP) bags for three and six months in Herat Afghanistan. Values are means± standard errors.

Storage Duration	Treatments	Moisture Content (%)	Adults of *T. castaneum*		Price/kg (AFA) *
			Alive	Dead	
0 month	Initial	8.28 ± 0.13 a **	0.00 ± 0.00 a	0.00 ± 0.00 a	18.90 ± 0.16 d
After 3 months	PICS bag	8.19 ± 0.12 a	0.00 ± 0.00 a	0.00 ± 0.00 a	22.00 ± 0.00 b
	PP bag	9.28 ± 0.22 b	3.00 ± 0.18 b	0.40 ± 0.12 a	19.45 ± 0.13 c
After 6 months	PICS bag	8.12 ± 0.12 a	0.00 ± 0.00 a	0.00 ± 0.00 a	25.00 ± 0.00 a
	PP bag	11.28 ± 0.12 c	10.05 ± 0.36 c	4.37 ± 0.304 b	14.20 ± 0.16 e
	ANOVA	F = 83.5; df = 4; *p* < 0.01	F = 602.2; df = 4; *p* < 0.01	F = 178.5; df = 4; *p* < 0.001	F = 1180.53; df = 4, *p* < 0.001

* Exchange rate: USD 1 = 60 Afghanis on 29 November 2013; ** means in the same column followed by the same letter are not significantly different (LSD, 5%).

**Table 3:** Assessment of the color and taste of bread made with wheat flour stored in Purdue Improved Crop Storage (PICS) and polypropylene (PP) bags for three and six months in Herat Afghanistan.

Storage Duration	Treatments		Color (% Respondents)				Taste (% Respondents)	
		Bad	Not Good	Fair	Good	Excellent	Not Good	Excellent
0 months	Initial	0	0	0	55	45	0	100
After 3 months	PICS bag	0	0	0	55	45	0	100
	PP bag	0	0	85	15	0	77.5	22.5
After 6 months	PICS bag	0	0	0	55	45	0	100
	PP bag	0	55	45	0	0	100	0

**Table 4:** Estimates return on investment (ROI) when wheat flour was stored for six months using 100 kg Purdue Improved Crop Storage (PICS) and polypropylene (PP) bags in Herat, Afghanistan. Grain and storage bag prices are in Afghani (AFA).

Storage Duration	Treatments	Price (AFA/100 kg Bag)		AFA				ROI *** (%)
		Initial	Later	Gross Margin	Price Bag *	OCC **	Net Gain	
After 3 months	PP bag	1.890	1.945	55.0	24	71.8	(40.8)	−2.1
	PICS bag	1.890	2.200	310.0	120	75.4	114.6	5.7
After 6 months	PP bag	1.890	1.420	(470.0)	24	143.6	(637.6)	−33.3
	PICS bag	1.890	2.200	610.0	120	150.8	339.3	16.9

* Price of (i) PICS bag is 120AFA and (ii) PP bag is 24 AFA (USD 1 = 60AFA on 29 November 2013). ** OCC: The opportunity cost of capital is estimated at 15% per year or 1.25% per month. Source: https://data.worldbank.org/indicator/FR.INR.LEND?locations=AF (accessed on 2 February 2022). *** ROI: Return on investments estimates are conservative because the cost is for one-season use (PICS bags can be used for 2 or 3 years).

## Data Availability

All data generated or analyzed during this study are included in this published article.
